# BIOMARKERS AND DRIVERS OF MITOCHONDRIAL HEALTH AND AGING

**DOI:** 10.1093/geroni/igae098.0984

**Published:** 2024-12-31

**Authors:** Anthony Molina

**Affiliations:** University of California San Diego, La Jolla, California, United States

## Abstract

In line with the principles of Geroscience, age-associated alterations in mitochondrial function have been shown to underlie a wide array of age-related diseases and conditions. This presentation will review mitochondrial bioenergetic profiling approaches that are being implemented in human/clinical studies. These include tissue and blood-based approaches that can serve as reliable biomarkers of mitochondrial health and have enabled researchers to examine the role of mitochondria in the physical and cognitive abilities of older adults. Importantly, we are also beginning to understand the factors that influence human mitochondrial bioenergetic capacity. These include intrinsic factors such as age and sex, as well as behavioral factors such as diet, exercise, and activity. We will also discuss ongoing efforts to identify circulating factors (e.g. proteins and metabolites) that drive differences in mitochondrial function associated with dementia and aging. Building upon our previous work demonstrating that blood-borne factors mediate age-related bioenergetic differences, our recent studies indicate that non-cellular factors present in serum can mediate bioenergetic differences associated with age and cognitive performance. Combining mitochondrial respirometry with multi-omic approaches, we are now able to identify circulating molecules that drive changes mitochondrial bioenergetics.

